# Comparative Analysis of the Kinematic Characteristics of Lunge-Style and Squat-Style Jerk Techniques in Elite Weightlifters

**DOI:** 10.3390/life14091086

**Published:** 2024-08-29

**Authors:** Gongju Liu, Zhanyang He, Binyong Ye, Haiying Guo, Huiju Pan, Houwei Zhu, Guanliang Meng

**Affiliations:** 1 Key Laboratory of Aquatic Sports Science, General Administration of Sport, Zhejiang College of Sports, Hangzhou 311200, China; liugongju@hotmail.com (G.L.); Ghy@zjcs.net.cn (H.G.); 2 College of Physical Education and Health Sciences, Zhejiang Normal University, Jinhua 321000, China; 2908161568@zjnu.edu.cn (Z.H.); binyongye@zjnu.edu.cn (B.Y.); panhuiju@zjnu.cn (H.P.)

**Keywords:** weightlifting, clean and jerk, kinematics and dynamics, human and bar combination barycenter

## Abstract

This study aimed to discuss the differences in technical characteristics between the lunge-style and squat-style jerk techniques and to reveal the adaptability of these techniques for individual weightlifters. A total of 52 attempts by 32 male weightlifters were selected, and the video data were digitized manually using the SIMI Motion 7.50 three-dimensional analysis system. The technical characteristics of the lunge split and squat jerk were fundamentally consistent during the pre-squat, force exertion, and inertia ascent phases. The primary differences between the lunge split and squat jerk techniques were observed during the squatting and support phases, including the vertical descent velocity of the barbell at the end of the squat shoulder-locking phase and the stability angles in the sagittal and coronal axes. The vertical velocity of the barbell at the end of the squat shoulder-locking phase was significantly greater in the squat style (−0.41 ± 0.17 vs. −0.88 ± 0.14) compared to the lunge style (*t* = 6.393, *p* < 0.05). The stability angle on the sagittal axis at the end of the squat-supporting phase in the lunge style was significantly greater (46.99 ± 3.23 vs. 13.64 ± 0.51) than that of the squat style (*t* = 45.639, *p* < 0.05).

## 1. Introduction

In weightlifting competitions, the final rank is based on the total score of the best successful snatch and clean and jerk [1]. The clean and jerk is the key to winning the gold medal: “Those who win the clean and jerk will conquer the weightlifting [2,3]”. The lunge-style jerk and the squat-style jerk are two types of jerk techniques in weightlifting [4], and there is an ongoing discussion regarding which technique is better. Some studies suggest that the squat-style jerk will replace the lunge-style jerk in the future [5]. In contrast, others believe that the squat-style jerk is not a widely applicable technique due to its lack of force economy principles (compared to athletes performing the lunge-style jerk, those performing the squat-style jerk exhibit a lower center of mass during the squat-supporting phase. As a result, during the standing-up phase, squat-style jerk athletes need to cover a greater vertical distance when applying force to the barbell.) poor stability, low success rate, long-term use leading to athlete coccyx disease, etc. [6,7]. Although the squat-style jerk technique has achieved great success in practice (as seen in the gold medal performances of Shi Zhiyong at the Rio and Tokyo Olympics), the debate over the pros and cons of squat-style and lunge-style jerk techniques still exists.

Analyzing the technical differences between the lunge-style jerk and squat-style jerk based on kinematic characteristics can contribute to innovations in weightlifting techniques and improve athletes’ performance. Previous studies have pointed out that the main differences between the lunge-style and squat-style jerk lie in the support and pushing stages [8]. In the lunge-style jerk, the support mainly relies on quickly separating the feet forwards and backward during the upward motion of the barbell at the end of the force phase and standing in an archery stance with the body’s vertical axis as the center and the feet in a front-back arrangement when catching the barbell. In contrast, in the squat-style jerk, the support relies on actively flexing the knees and hips and pushing up the barbell at the end of the force phase [9]. Previous research based on the principles of biomechanics analyzed the advantages and disadvantages of the two techniques, pointing out the advantages of the squat-style jerk technique, including allowing for deeper squatting during the catching stage, a lower vertical height of the barbell, and greater potential for lifting heavier weights [8]. However, its disadvantages include a smaller support surface for the feet in the sagittal plane, a lower success rate, and increased difficulty in pushing up the barbell due to the body’s lower center of gravity (COG) during the squatting phase. The advantages of the lunge-style jerk technique are a larger stable angle during the catching stage, better barbell supporting stability, and ease in pushing up the barbell [5,6,7,10,11]. However, although previous studies have analyzed the squat-style and lunge-style jerk techniques based on the principles of biomechanics with small sample sizes of athletes, significant individual differences exist in the technical characteristics of top elite athletes, necessitating further large-scale samples to verify the findings.

Currently, the number of athletes using the squat jerk technique in international Olympic weightlifting competitions is relatively small, with the majority being Chinese athletes. This makes it challenging to compare the differences between the lunge jerk and squat jerk techniques using large sample sizes. In this study, we aim to analyze the differences between the lunge jerk and squat jerk techniques among elite athletes using as large a sample as possible. We employ kinematic analysis methods to compare joint angles, barbell displacement and velocity, and center of mass displacement of elite athletes using the lunge and squat jerk techniques in major competitions. Our goal is to gain a deeper understanding of the technical differences between the two jerk techniques, provide theoretical support for the selection and development of weightlifting athletes and techniques, and offer technological assurance for the innovation of weightlifting training. Compared to the squat-style jerk, the lunge-style jerk has a larger contact area of the lower limbs on the sagittal plane during the support phase, but a smaller contact area on the coronal plane. Therefore, we hypothesize that the lunge-style jerk will have a larger stability angle in the sagittal plane and a smaller stability angle in the coronal plane during the support phase. Additionally, during the squat shoulder locking phase, the vertical descent speed of the barbell’s center of mass in the squat-style jerk is expected to be faster than in the lunge-style jerk.

## 2. Methods

### 2.1. Subjects

Data from national and international adult men’s weightlifting competitions recorded by the research team since the third level adjustment in 2018 were selected and compiled. This study organized data from major national and international men’s weightlifting competitions recorded by the research team since 2018 (following the International Weightlifting Federation’s (IWF) third-level adjustment on 6 July 2018). After the screening, only 20 successful squat jerk attempts (height: 169.90 ± 3.45 cm, weight: 74.21 ± 9.51 kg) and 26 successful lunge jerk attempts (height: 168.00 ± 4.14 cm, weight: 76.21 ± 8.65 kg) met the screening criteria: ((1). The competition must be a Chinese national or international event after 2018; (2). The selected athletes must be elite-level (Chinese national master level, won top three in Chinese national, world, and Olympic weightlifting competitions, member of the Chinese national team.); (3). The successful lift must be the athlete’s maximum successful lift in the competition; (4). The selected weight categories are 61, 67, 73, and 81 kg).

All selected samples are world-class high-level athletes, from the China Weightlifting Championships, Olympic Qualifying Tournaments, Asian Championships, and World Championships, with results reaching international standards and having good representativeness. This study is based on the sports science projects of the Department of Science and Technology of Zhejiang Province. Its contents and methods have been determined to be legally valid and approved by the Ethics Committee of the Zhejiang Normal University (No. ZSRT2023078). All video data are from open competitions without intervention and the video capture method used did not affect the athletes’ performance in any way.

### 2.2. Procedures

#### 2.2.1. Data Collection

**Figure 1:**
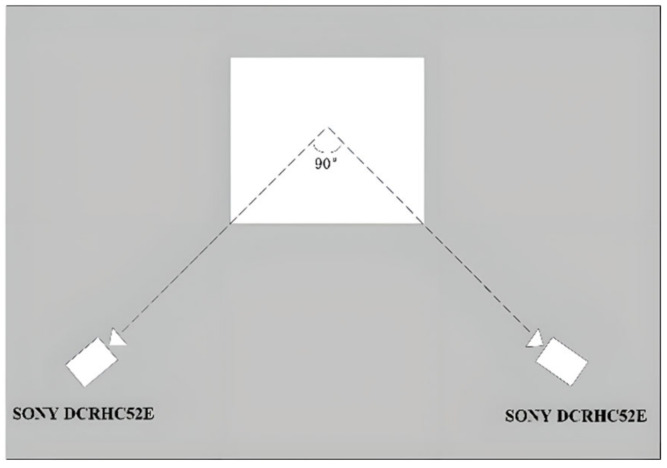
Schematic diagram for the setting of the camera system.

**Figure 2:**
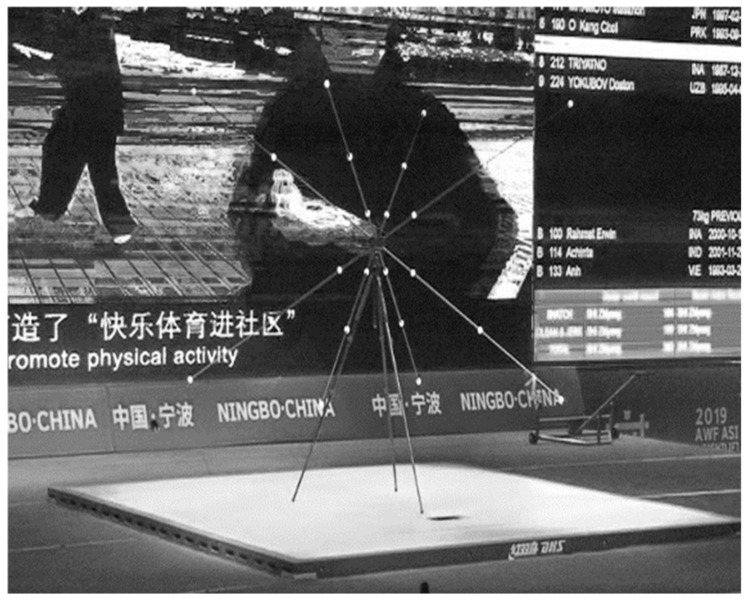
Setting of the 24-point peak frame for three-dimensional space.

Camera System: Two SONY NEX-VG900E cameras (Sony Corporation, Tokyo, Japan)(lens: SONY SEL18200LE) were used to record the competition at a frame rate of 50 frames per second. Two cameras were positioned on the left and right sides of the lifting platform, with an angle of approximately 90° between their main optical axes centered on the platform. The cameras were placed at a distance of about 10 m from the subjects, and both the camera positions and focal lengths were kept constant throughout the experiment [12] (see Figure 1 and Figure 2).

Coordinate System: before the competition, a 24-point peak frame was placed on the competition venue, and the three-dimensional spatial calibration of the venue was carried out (see Figure 2). The coordinate system was set as the positive *X*-axis direction pointed directly behind the athlete, the positive *Y*-axis direction pointed to the athlete’s right side, and the positive *Z*-axis direction was vertically upward.

#### 2.2.2. Data Analysis and Calculation

Data analysis: The German SIMI Motion10.2 (SIMI Reality Motion Systems GmbH, Munich, Germany) three-dimensional analysis system was used to digitize the lifting process, with a parsing frequency of 50 Hz. The video data were intercepted from the first four frames after the barbell lift-off and then manually processed frame by frame. Seventeen key anatomical landmarks were established for lifting technique digitalization analysis, including the head, left and right shoulder joint centers, left and right elbow joint centers, left and right wrist joint centers, left and right hip joint centers, left and right knee joint centers, left and right ankle joint centers, left and right toe tips, and the left and right endpoints of the barbell [12]. The COG of the human body was calculated based on Hanavan’s mathematical model of the human body. The geometric center point of the barbell (the center point between the two endpoints) was used as its COG position. The spatial coordinates of the human body were obtained using the DLT (Direct Linear Transform) calculation method, and relevant kinematic parameters were calculated [13,14]. The raw position-time data were smoothed by a low-pass digital filter with a cut-off frequency of 4 Hz [15,16]. 

Division of jerk phases: The jerk action can be divided into six stages: preparation, pre-squat (active pre-squat stage and pre-squat braking stage), force, inertial rising, squat supporting, and standing up [8,17,18,19]. To gain a deeper understanding of the technical differences between the squat-style jerk and lunge-style jerk, we subdivided the jerk process into more detailed stages, drawing on the framework established in previous studies. The jerk action can be divided as follows: the first part is from the start of preparation to the end of the force stage, and the second part is from the inertial rising to the end of standing up (Table 1). In addition, the lunge-style jerk athletes used a lunge-step squat-supporting barbell during the inertial rising phase and the squat-supporting phase, and either their left or right foot was in front, presenting a lunge-step posture. Therefore, they were named “front hip and knee joint angle” and “back hip and knee joint angle”. The squat-style jerk athletes use a double-legged squat posture, so there is no distinction between front and back legs, and the joint angles on both sides are essentially the same. In the comparative analysis, the right foot of the squat-style jerk is compared with the front foot of the lunge-style jerk, and the right foot of the squat-style jerk is compared with the back foot of the lunge-style jerk.

### 2.3. Research Variable

Differences in the technical characteristics of the lunge-style and squat-style jerk were identified by comparing parameters such as time duration, hip joint angle, knee joint angle, the vertical height of the barbell, the vertical velocity of the barbell, and the distance between the “two centers” on the X, Y, and Z axes (the absolute value of the difference between the COG of the human body and the barbell) of each stage in the first part of the jerk action [20,21]. In addition, to further investigate the technical differences between the lunge-style and squat-style jerk during the squat-supporting phase, the present study compared the vertical changes in the barbell, grip distance, X-axis stance distance (the distance from the front foot toes to the back foot heels on the X-axis direction), Y-axis stance distance (the distance between the inner edges of both feet on the Y-axis), X-axis stable angle (the angle between the COG of the barbell and the front foot toes and back foot heels in the sagittal plane), Y-axis stable angle (the angle between the COG of the barbell and the center of the outer edge of both feet in the coronal plane), maximum acceleration of the barbell during the M11-2, and the angle between the arm and the barbell. 

### 2.4. Statistical Analyses

All data were presented as mean ± SD. Levene’s test was used to test the homogeneity of variance, and the Kolmogorov-Smirnov test was used to test the normality of the data. The obtained data were classified and arranged, and an independent sample *t*-test was used to compare the technical parameters of the lunge-style and squat-style jerk. A paired samples *t*-test was employed to analyze the technical parameters of successful and unsuccessful attempts in both the lunge-style and squat-style jerks. SPSS 24.0 software (IBM, Armonk, NY, USA) was used for data analysis and the significance level was set at *p* < 0.05. Cohen’s *d* was used to evaluate effect sizes (ES), which were defined as small (0–0.19), medium (0.20–0.49), large (0.50–0.79), and very large (≥0.80) [22].

## 3. Results

### 3.1. Comparison of Stage Parameters between the Lunge and Squat Styles in the First Part of the Jerk Action

As shown in Table 2, during the pre-squat stage, the time duration of the active pre-squat stage (M8-1) of the lunge-style jerk lasted 0.23 s, which was significantly longer than that of the squat-style jerk (*t* = 3.131, *p* < 0.05, ES = 1.018). The hip joint angles of both sides at the end of the pre-squat braking stage (M8-2) for the lunge-style jerk were significantly higher than those of the squat-style jerk (*t* = 2.641, *p* < 0.05, ES= 0.838; *t* = 2.714, *p* < 0.05, ES = 0.858). During the active pre-squat and the pre-squat braking stages, the distances between the “two centers” on the *Z*-axis of the lunge-style jerk were significantly lower than those of the squat-style jerk (*t* = −2.720, *p* < 0.05, ES = 0.871; *t* = −3.123, *p* < 0.05, ES = 0.980). 

During the force stage (M9), the knee and hip joint angles on both sides of the squat-style jerk were smaller than those of the lunge-style jerk at the end of the force stage. Specifically, the hip joint angles of the lunge-style jerk were statistically significantly higher than those of the squat-style jerk (*t* = 2.021, *p* = 0.05, ES = 0.645; *t* = 2.111, *p* < 0.05, ES = 0.667). The knee joint angles of the lunge-style jerk were significantly higher than those of the squat-style jerk (*t* = 2.113, *p* < 0.05, ES = 0.698; *t* = 2.034, *p* < 0.05, ES = 0.643). The distances between the “two centers” of the lunge-style jerk on the *X*-axis, *Y*-axis, and *Z*-axis represented statistically significant differences (*t* = 3.185, *p* < 0.05, ES = 0.996; *t* = 2.678, *p* < 0.05, ES = 0.834; *t* = −2.586, *p* < 0.05, ES = 0.828). 

### 3.2. Comparison of the Stage Parameters between the Lunge and Squat Styles in the Second Part of the Jerk Action

Differences in the technical characteristics between the lunge-style and squat-style jerk in each stage of the second part of the jerk action are shown in Table 2. In the inertia-rising stage (M10), the hip and knee joint angles of the front leg for the lunge-style jerk showed significant differences (*t* = −2.747, *p* < 0.05, ES = 0.896; *t* = 2.624, *p* < 0.05, ES = 0.852). The hip and knee joint angles of the back leg for the lunge-style jerk showed statistically significant differences (*t* = 6.689, *p* < 0.05, ES = 2.16; *t* = 14.596, *p* < 0.05, ES = 4.569). 

In the squat shoulder locking stage (M11-1), the differences in the “two-center” distance between the two groups on the X, Y, and Z axes were not statistically significant. Differences in all other parameters were statistically significant. The time duration of the lunge-style jerk was 0.11 s, which was 0.04 s less than that of the squat-style jerk, representing statistically significant differences (*t* = −2.269, *p* < 0.05, ES = 0.667). The hip and knee joint angles of both sides for the lunge-style jerk were greater than those for the squat-style jerk. The hip joint angles for the lunge-style jerk were greater (by 9.01° and 60.09°) than those of the squat-style jerk, representing statistically significant differences (*t* = 3.41, *p* < 0.05, ES = 1.09; *t* = 20.701, *p* < 0.05, ES = 6.889). The knee joint angles for the lunge-style jerk were greater (by 26.53° and 59.05°) than those for the squat-style jerk, with statistically significant differences (*t* = 9.954, *p* < 0.05, ES= 3.242; *t* = 16.557, *p* < 0.05, ES = 5.158). The vertical height of the barbell for the lunge-style jerk was 4.80 cm higher than that of the squat-style jerk, representing a statistically significant difference (*t* = 3.915, *p* < 0.05, ES= 0.796). The vertical falling velocity of the barbell in the squat-style jerk was 0.47 m/s faster than that of the lunge-style jerk, denoting a statistically significant difference (*t* = 6.393, *p* < 0.05, ES = 2.98).

At the end of the active supporting stage (M11-2), the differences in the distance of the “two centers” on the *X* and *Y* axes between the two groups were not statistically significant. All other parameters showed statistically significant differences. The time duration for the lunge-style jerk of the active supporting phase was 0.19 s, which was 0.39 s less than that of the squat-style jerk, representing a statistically significant difference (*t* = −7.574, *p* < 0.05, ES = 2.59). The hip and knee joint angles of both sides for the lunge-style jerk were greater than those of the squat-style jerk. The hip joint angles for the lunge-style jerk were greater (by 42.92° and 100.25°) than those of the squat-style jerk and the differences were statistically significant (*t* = 15.737, *p* < 0.05, ES = 4.871; *t* = 40.647, *p* < 0.05, ES = 12.679). The knee joint angles for the lunge-style jerk were greater (by 58.42° and 99.72°) than those for the squat-style jerk, indicating statistically significant differences (*t* = 22.499, *p* < 0.05, ES= 7.643; *t* = 33.086, *p* < 0.05, ES = 10.18). The vertical height of the barbell in the lunge-style jerk was 24.29 cm higher than that in the squat-style jerk, representing statistically significant differences (*t* = 18.876, *p* < 0.05, ES = 4.661). The “two-center” distance on the *Z*-axis for the lunge-style jerk was 2.98 cm greater than that for the squat-style jerk, displaying a statistically significant difference (*t* = 3.199, *p* < 0.05, ES = 1.021).

### 3.3. Comparison of Stability Factors in the Second Phase of the Jerk between Athletes Using the Lunge Jerk and Squat Jerk Techniques

**Figure 3:**
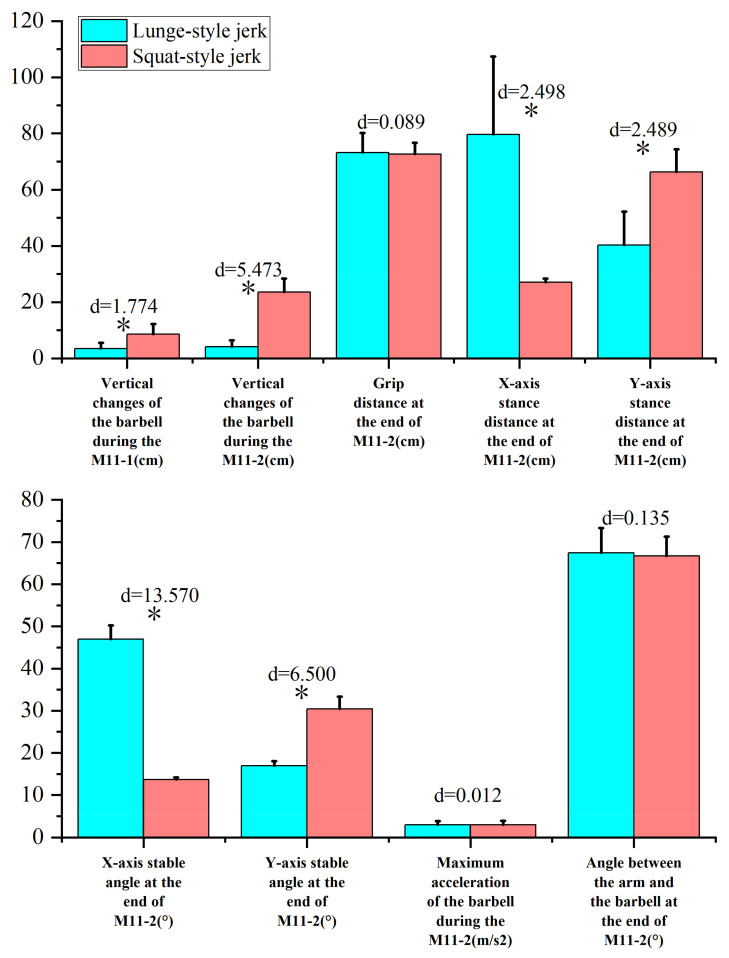
Further comparison of the technical characteristics in the squat-supporting phase between the lunge-style and squat-style jerks. * represents *p* < 0.05.

There are significant differences in the vertical changes in the barbell during the squat shoulder locking phase and active supporting phase (Figure 3). In the squat shoulder locking stage (M11-1), the vertical change in the barbell for the lunge-style jerk was 3.49 cm, which was 5.06 cm less than that for the squat-style jerk, representing a statistically significant difference (*t* = −5.42, *p* < 0.05, ES = 1.774). In the active supporting phase (M11-2), the vertical change in the barbell for the lunge-style jerk was 4.12 cm, which was 19.48 cm less than that for the squat-style jerk, displaying a statistically significant difference (*t* = −16.562, *p* < 0.05, ES = 5.473). At the end of the active supporting phase, the *X*-axis stance distance of the lunge-style jerk was 52.51 cm larger than that of the squat-style jerk (*t* = 19.086, *p* < 0.05, ES = 2.498), while the *Y*-axis stance distance of the former was 25.98 cm smaller than that of the latter (*t* = −7.700, *p* < 0.05, ES = 2.489). The *X*-axis stable angle of the former was 33.35° larger than that of the latter (*t* = 45.639, *p* < 0.05, ES = 13.570), while the *Y*-axis stable angle of the former was 13.49° smaller than that of the latter (*t* = −19.47, *p* < 0.05, ES = 6.500). There were no statistically significant differences in the grip distance, the angle between the arm and barbell at the end of the squat-supporting phase, or the maximum acceleration of the barbell during the M11-2 stage between the lunge-style jerk and squat-style jerk.

## 4. Discussion

In this study, we employed kinematic analysis methods to compare the joint angles, barbell displacement and velocity, and center of mass displacement in elite athletes using the lunge-style and squat-style jerk techniques during major competitions. The goal of this research is to identify the technical differences between the two techniques, providing theoretical support for the selection and development of weightlifting athletes, as well as offering technological assurance for the innovation of weightlifting training. The jerk action in weightlifting can be divided into two parts: the first part consists of the pre-squat phase (active pre-squat phase and pre-squat braking phase) and the force phase, which involves utilizing the ground reaction force to accelerate the barbell through a pre-squat, and extending the knee and hip joint and pulling the barbell quickly in the force phase so that the barbell can obtain the appropriate initial speed at the end of the first part [7,9,23]. The second part consists of the inertial rising, squat-supporting (squat shoulder locking stage and active supporting stage), and standing-up stages. The initial speed generated in the first phase allows the barbell to continue rising due to inertia. Simultaneously, the barbell can maintain a certain velocity as it is supported by the extended arms during its ascent. At the same time, athletes split or bend their legs quickly to complete the supporting barbell, and then stand up with the barbell to complete the whole jerk action [24].

In the pre-squat phase (M8) of jerk action, the pre-squat phase mainly involves bending the knees and hip, lowering the COG of the human body so that the human body and barbell accelerate first (active pre-squat), and then decelerating (pre-squat braking). The velocity of the barbell at the end of the active pre-squat phase and the time duration of the pre-squat barking phase are the main factors affecting the effectiveness (the elastic potential energy stored in the barbell) of the pre-squat action [7,9,23]. According to the law of the momentum principle, the momentum generated by the barbell during the active pre-squat phase is equal to the impulse generated during the pre-squat braking phase, *mv2* − *mv1* = *F* × Δ*t* (Equation (1)), where *v2* is the velocity at the end of the active pre-squat phase, and *v1* is the initial velocity of 0. Thus, *mv^2^* = *F* × Δ*t* (Equation (2)). The greater the velocity at the end of the active pre-squat phase, the greater the momentum of the active pre-squat phase and the impulse of the pre-squat braking phase. The momentum is translated into maximum impact force, which acts on the ground generating a reactive force that causes elastic deformation of the barbell through the human body. Following Equation (2), when the *mv^2^* is constant, *F* and Δ*t* are inversely proportional. The time duration of the pre-squat braking phase reflects the ability to transform from eccentric contraction to concentric contraction under nearly extreme load conditions. A shorter braking time results in a stronger impact force, greater elastic deformation of the barbell, and better upward jerk performance [10,11]. By comparing the technical characteristics of the lunge-style and squat-style jerk, the present study found that the velocity at the end of the active pre-squat phase and the time duration of the pre-squat braking phase varied slightly between the two groups. Only the time duration of the active pre-squat phase in the squat-style jerk was shorter than that in the lunge-style jerk. Additionally, studies by Ai et al. [6] and Wu et al. [14] suggested that a reasonable braking time for the lunge-style jerk is about one-third of the entire pre-squat period, while the braking time for the squat-style jerk is about half the pre-squat period. The findings of the present study are consistent with those of previous research.

In the force phase (M9), the barbell can rise faster by extending the knee and hip joints, as well as flexing the ankles, contracting the glutes, and extending the arms, so that the barbell can achieve a greater initial velocity beginning the inertia-rising phase [18]. Kipp et al. [25] pointed out that a sufficient extension of the knee and hip joints during the force phase, as well as the vertical velocity of the barbell at the end of this phase, are critical for the barbell to reach the necessary height at the end of the inertia-rising phase. The present study compared the technical parameters of the force phase between the lunge-style and squat-style jerk. The vertical velocity of the barbell at the end of the force phase differed by only 0.01 m/s between the two jerks. The squat-style jerk had smaller hip and knee joint angles than the lunge-style jerk on both sides, and the vertical distance of the “two centers” in the squat-style jerk was larger than that in the lunge-style jerk. These results indicate that in the lunge-style jerk, to achieve a greater vertical velocity of the barbell in the force phase, the leg and hip extension are greater than those in the squat-style jerk, resulting in a higher vertical position of the COG of the human body. Therefore, when the COG of the barbell is similar, the “two centers” in the lunge-style jerk on the *Z*-axis are closer than those in the squat-style jerk. To fully extend the knee and hip joints, the COG of the human body in the lunge-style jerk will shift backward, resulting in a larger distance of the “two centers” on the *X*-axis at the end of the force phase compared to that in the squat-style jerk. Additionally, due to the larger amplitude variations of the human body in the lunge-style jerk, the distance of the “two centers” on the *Y*-axis at the end of this phase is also higher than that in the squat-style jerk. Moreover, the results of a related study [19] on the large difference in vertical velocity of the barbell at the end of the force phase between female lunge-style jerk and squat-style jerk are inconsistent with our results. This may be due to differences in the gender distribution or small sample sizes in the previous study.

During the inertia-rising phase (M10), to complete the supporting action in subsequent split feet or squatting techniques, the barbell must be lifted to the appropriate height, and the vertical height of the barbell rising is one of the key factors for a successful lift [14]. In this study, the vertical height of the barbell at the end of the inertia-rising phase for the lunge-style jerk was 155.22 cm, which was only 0.28 cm higher than that of the squat-style jerk. Furthermore, this study found that at the beginning of the inertia-rising phase, the vertical velocities of the barbell of the lunge-style and squat-style jerk were 1.52 m/s and 1.53 m/s, respectively, which according to the laws of momentum and energy, could only lift the barbell by about 12 cm. However, both jerk types lifted the barbell over 20 cm during the inertia-rising phase, indicating that athletes must continue to apply an upward force on the barbell during this phase.

**Figure 4:**
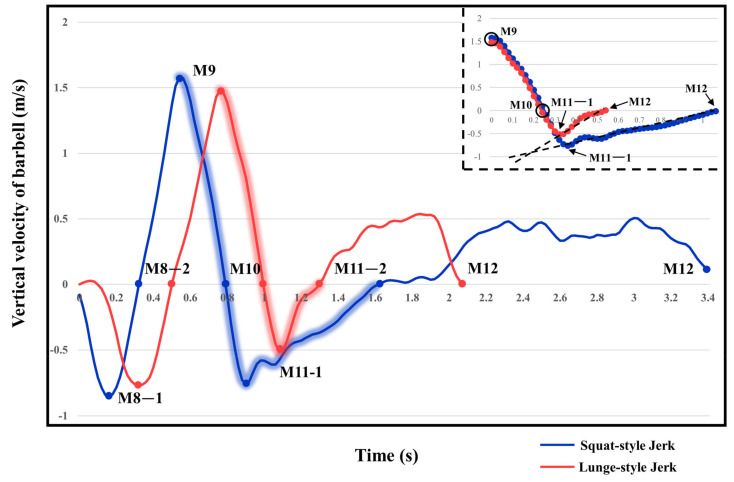
Changes in the vertical velocity of the barbell in successful attempts of the lunge-style and squat-style jerks.

Based on the trend of the vertical velocity of the barbell during the squat-supporting phase (M11), this study divided the M11 phase into two stages: the squat shoulder locking phase (M11-1) and the active supporting phase (M11-2). The squat shoulder locking phase (M11-1) refers to the period from the moment of the first peak in the vertical height of the barbell until the maximum falling velocity of the barbell. During this stage, the athlete’s arms are basically in a straight state, and they will continue to squat slightly to fully lock the shoulder joint. At this stage, the COG of the human body drops by 5.14 cm in the lunge-style jerk and 10.61 cm in the squat-style jerk. Additionally, by actively squatting and locking the shoulder joint to increase the vertical distance of the COG of the human body and barbell while continuously increasing the strength of the arm supporting the barbell, the acceleration of the barbell decreases continuously. As the force applied by the athlete on the barbell is smaller than the weight of the barbell at this stage, the vertical falling velocity of the barbell gradually increases until it reaches a maximum when the force applied by the athlete equals the weight of the barbell. Since the squat-style jerk spends more time in the squat shoulder locking stage, the vertical downward velocity of its barbell is larger, reaching −0.88 m/s, compared with only −0.41 m/s in the lunge-style jerk (Figure 4).

The active supporting stage (M11-2) refers to the period from the moment of the maximum falling velocity of the barbell to the moment when the barbell falls back to its lowest point. At this stage, the force applied by the athlete on the barbell is greater than the barbell weight, and the vertical velocity of the barbell gradually decreases from its maximum to zero. During this stage, the COG of the human body and barbell in the lunge-style jerk only drop slightly, by 1.59 cm and 4.11 cm, respectively. In the squat-style jerk, they drop greatly by 17.29 cm and 23.60 cm, respectively. The primary reason that the lunge-style jerk is considered superior to the squat-style jerk is due to the shorter distance the barbell travels during the descent phase in the lunge position. This results in a smaller impact force when the barbell is caught, as well as a shorter distance over which the athlete needs to exert force during the upward drive. Consequently, the energy expenditure required to control the barbell is reduced. In contrast, athletes performing the squat-style jerk spend more time and cover a greater distance when supporting the barbell during the drive phase, leading to significant differences in the work done and the average force exerted between the two techniques. According to the law of conservation of energy, the work done by the athlete on the barbell *W* = *F* × *S* (Equation (3)), where *F* is the force applied by the athlete and *S* is the distance over which the force is applied) is equal to the sum of the kinetic energy at the beginning of the drive phase and the difference in potential energy from the start to the end of the phase, *W* = *mg*Δ*h* + 1/2 *mv*^2^ (Equation (4)), where *m* is the mass of the barbell, Δ*h* is the vertical displacement of the barbell during the drive phase, and *v* is the initial vertical descent speed of the barbell). Therefore, *F* × *S* = *mgh* + 1/2 *mv*^2^ (Equation (5)). Through this derivation, it can be concluded that the average force exerted by squat-style jerk athletes is lower than that of lunge-style jerk athletes. As a result, when comparing the squat-style and lunge-style jerks, it is crucial to consider which factor—total work done or average force—is more challenging to improve during the drive phase. Moon’s research suggests that for world-class athletes, physical fitness and muscle strength are relatively difficult to enhance while improving weightlifting technique is comparatively easier [26]. Therefore, in order to increase the success rate in lifting attempts, it is essential to determine whether improving strength or technique is more challenging in the context of the lunge-style and squat-style jerks. This study suggests that, at near-maximal weights, improving the strength required for the lunge-style jerk is significantly more difficult than optimizing the technique for the squat-style jerk. As a result, the squat-style jerk may gradually become the dominant technique in the future.

Additionally, due to variations in the squatting posture at the end of the squat-supporting phase, there are noticeable differences in the forward-backward and left-right stance distances, as well as in the stability angles in all directions between the two jerk types. Our study found that the sagittal plane stability angle for the squat-style jerk is 13.64°, which is 33.35° smaller than that of the lunge-style jerk. These findings support our research hypotheses: (1) the lunge-style jerk exhibits a greater stability angle in the sagittal plane during the support phase, and (2) the squat-style jerk demonstrates a larger stability angle in the coronal plane. This is consistent with previous studies [19] which concluded that the sagittal plane stability angle in the squat-style jerk is significantly smaller than that in the lunge-style jerk. Additionally, our study observed that the left-right stance distance in the squat-style jerk is greater than that in the lunge-style jerk. Furthermore, the coronal plane stability angle in the squat-style jerk is 30.44°, which is 13.49° greater than that in the lunge-style jerk. Compared to the lunge-style support, the squat-style support provides a similarly adequate stability angle in the coronal plane. However, in the sagittal plane, the stability angle for the squat-style support is significantly smaller, resulting in reduced overall stability. This makes it more challenging to maintain balance, as even a slight deviation can lead to forward or backward tilting, making it difficult to adjust the support position in time to complete the lift. The squat-style support demands exceptionally strong core muscles, coordination, and flexibility from the athlete. In contrast, the lunge-style support, with its wider stability angle due to the split stance, offers greater stability. This allows the athlete to more easily adjust their posture and successfully complete the lift even if the center of mass shifts during the movement.

During the standing-up phase (M12), in this study, the vertical distance of the COG of the barbell in the squat-style jerk was 54.55 cm, which is 22.84 cm greater than that in the lunge-style jerk. For the squat-style jerk, the vertical distance to be traveled by the COG of the barbell is longer, and it takes more time and energy in the standing-up phase, which requires higher leg strength, lower limb joint flexibility, and core control ability. These findings are consistent with those of previous studies [27,28,29].

## 5. Limitations

All video data were captured under competition conditions, due to the location of TV broadcast cameras and the environment of the competition site, we only used two cameras for video shooting. In video parsing, it is common that body joints are hidden by local limbs or not visible on the side camera. Fortunately, the staff who processed video data is familiar with human anatomy, and they have more than fifteen years of experience using SIMI°Motion7.50 3D analysis software. These facts guarantee the accuracy of the data. Our sample primarily consists of athletes from the Chinese Weightlifting Championships, Olympic Trials, Asian Championships, and World Championships—participants who are highly experienced and have undergone extensive training. Due to the sample size, specific population focus, gender, and individual differences among athletes, the findings of this study are most applicable to high-level male athletes with similar backgrounds. Although this limits the generalizability of our conclusions, the results still offer valuable insights into the performance patterns of world-class weightlifters.

## 6. Conclusions

The technical characteristics of the lunge-style jerk and squat-style jerk are similar in the pre-squat, force, and inertial rising phases. The primary differences between the two styles are observed during the squat supporting phase, including confirmed joint angle changes, time duration, and vertical height of the barbell at the end of the phase. Additionally, there are significant differences in the vertical falling velocity of the barbell and the stable angle on the sagittal and coronal axis in the squat shoulder locking phase. Although the lunge-style and squat-style jerks use different power generation methods, both achieve the desired power and require adequate barbell height. The squat-style jerk demands faster hip flexion and higher technical stability, while the lunge-style jerk requires greater strength. With extreme mass conditions, enhancing lunge-style jerk strength is harder than optimizing the squat-style jerk technique, indicating the squat-style jerk might become more common. This study advances our understanding of kinematic variables for both jerk styles, but further research is needed. Future studies could use techniques like principal component analysis and unsupervised machine learning to explore kinematic predictors of lift success, offering more precise guidance for athletes and coaches.

## Figures and Tables

**Table 1:** Division of phases of lunge-style and squat-style jerks.

Phase	Lunge-Style Jerk	Squat-Style Jerk
M7: Preparation phase From the end of the clean action to the beginning of the squatting knee flexion	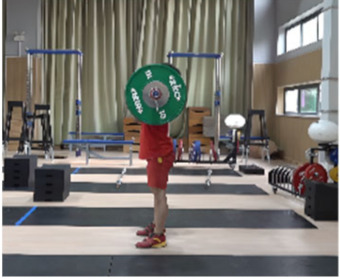	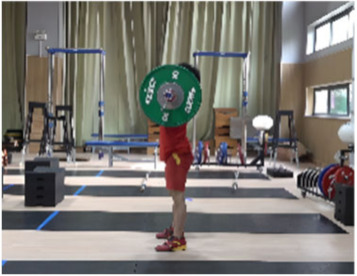
M8: pre-squat phase (From the beginning of the squatting knee flexion to the moment of the smallest knee joint angle)		
M8-1: Active pre-squat stage From the onset of pre-squat to the moment of maximum barbell falling velocity	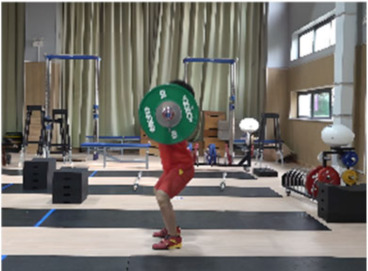	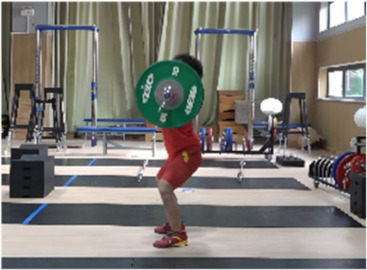
M8-2: Pre-squat braking stage From the moment of maximum barbell falling velocity to the moment of the smallest knee joint angle	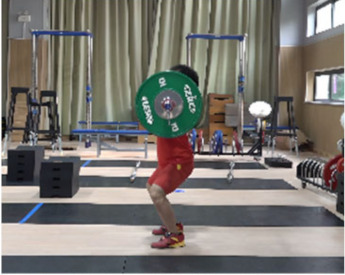	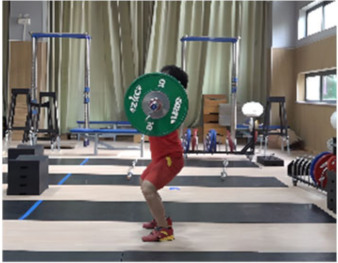
M9: Force phase From the moment of the smallest knee joint angle to the moment of maximum vertical velocity of barbell	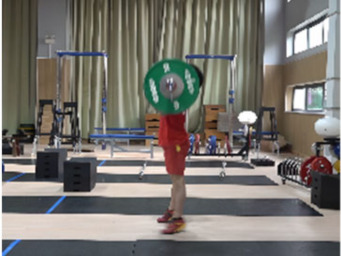	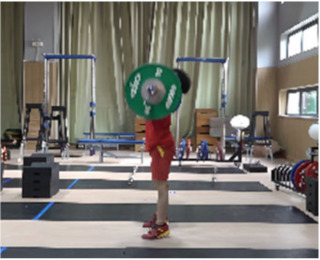
M10: Inertial rising phase From the moment of the maximum vertical velocity of the barbell to the moment of the first peak value of the vertical height of the barbell	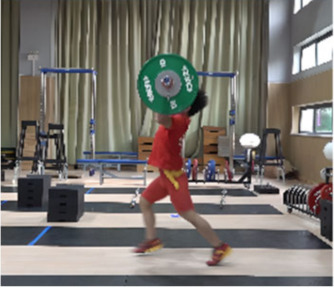	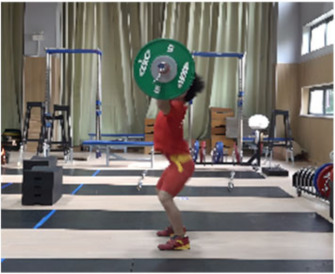
M11: Squat-supporting phase (From the moment of the first peak value of the barbell vertical height to the moment of the lowest point of barbell falls again)		
M11-1: Squat shoulder locking stage From the moment of the first peak value of the barbell vertical height to the moment of maximum barbell falling velocity	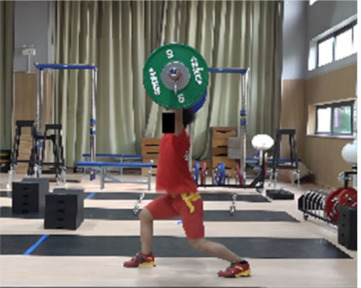	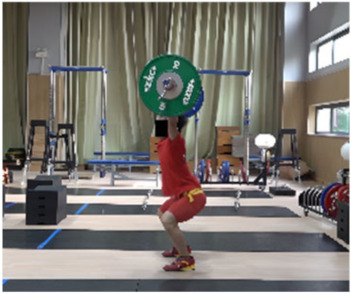
M11-2: Active supporting stage From the moment of maximum barbell falling velocity to the moment of the lowest point of barbell falls again	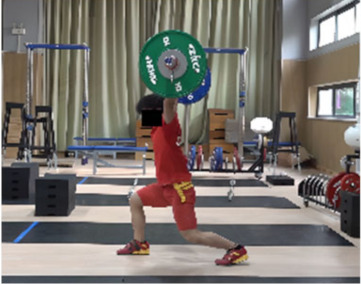	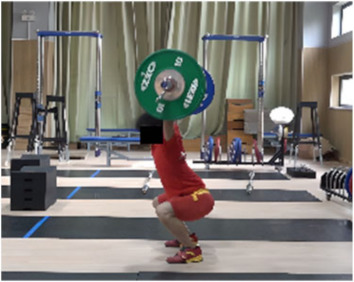
M12: Standing-up phase From the moment of the lowest point of the barbell falls again to the second peak value of the barbell vertical height	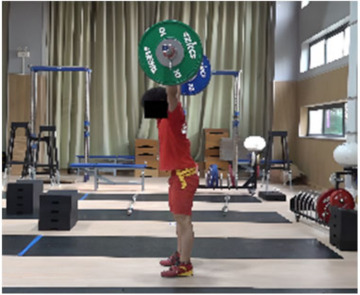	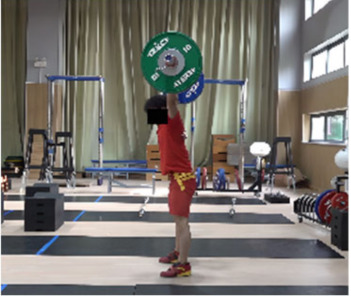

**Table 2:** Comparison of technical characteristics across phases in the lunge-style and squat-style jerks.

		End of M7	M8		End of M9	End of M10	M11		End of M12
			End of M8-1	End of M8-2			End of M11-1	End of M11-2	
Time duration(s)	Lunge style	2.57 ± 2.37	0.23 ± 0.07	0.15 ± 0.04	0.25 ± 0.04	0.23 ± 0.02	0.11 ± 0.06	0.19 ± 0.08	1.34 ± 0.40
	Squat-style	3.19 ± 1.65	0.17 ± 0.04	0.16 ± 0.04	0.25 ± 0.04	0.22 ± 0.02	0.15 ± 0.06	0.58 ± 0.21	1.93 ± 0.75
	*p*-value	0.341	0.003	0.488	1.00	0.496	0.029	<0.001	0.03
	Cohen’s *d*	0.297	1.018	0.250	0	0.5	0.667	2.59	1.021
Front hip joint angle (°)	Lunge style	162.21 ± 8.48	141.04 ± 6.57	134.83 ± 7.31	161.80 ± 6.80	114.71 ± 7.06	99.76 ± 7.68	94.76 ± 9.9	168.13 ± 6.58
	Squat-style	158.27 ± 6.11	138.86 ± 7.09	128.52 ± 7.82	157.44 ± 6.71	122.95 ± 11.41	90.75 ± 8.98	51.84 ± 7.13	155.55 ± 6.96
	*p*-value	0.100	0.318	0.012	0.050	0.009	0.002	<0.001	0.139
	Cohen’s *d*	0.522	0.321	0.838	0.645	0.896	1.09	4.871	1.865
Back hip joint angle (°)	Lunge style	162.64 ± 9.13	141.52 ± 6.95	134.84 ± 7.74	162.07 ± 7.11	143.18 ± 8.23	150.65 ± 8.49	151.90 ± 8.55	159.12 ± 7.68
	Squat-style	158.77 ± 6.48	138.56 ± 6.98	128.23 ± 7.65	157.37 ± 6.96	122.40 ± 11.19	90.56 ± 9.02	51.65 ± 6.97	155.15 ± 6.37
	*p*-value	0.159	0.186	0.010	0.041	0.000	<0.001	<0.001	0.083
	Cohen’s *d*	0.478	0.425	0.858	0.667	2.16	6.889	12.679	0.556
Front knee joint angle (°)	Lunge style	163.01 ± 10.98	119.77 ± 5.44	106.4 ± 5.62	168.50 ± 6.47	109.30 ± 6.22	103.88 ± 6.40	102.06 ± 7.88	160.88 ± 9.00
	Squat-style	159.14 ± 4.25	121.2 ± 6.61	110.2 ± 11.38	163.91 ± 6.72	102.86 ± 9.03	77.35 ± 10.06	43.64 ± 7.32	159.75 ± 8.86
	*p*-value	0.150	0.458	0.188	0.041	0.012	<0.001	<0.001	0.693
	Cohen’*s d*	0.443	0.239	0.442	0.698	0.852	3.242	7.643	0.126
Back knee joint angle (°)	Lunge-style	162.95 ± 11.16	120.16 ± 6.24	106.75 ± 6.31	168.01 ± 6.8	147.12 ± 10.31	136.22 ± 12.48	143.28 ± 11.35	160.13 ± 11.00
	Squat-style	159.37 ± 4.76	121.68 ± 6.14	110.28 ± 11.12	163.67 ± 6.69	102.77 ± 8.85	77.17 ± 9.93	43.56 ± 7.26	160.48 ± 9.23
	*p* value	0.195	0.442	0.225	0.049	<0.001	<0.001	<0.001	0.915
	Cohen’*s d*	0.399	0.245	0.405	0.643	4.569	5.158	10.18	0.034
Vertical height of barbell (cm)	Lunge-style	132.27 ± 2.73	123.04 ± 2.76	114.05 ± 2.82	134.29 ± 2.38	155.22 ± 5.92	151.46 ± 4.83	147.35 ± 5.87	179.06 ± 5.21
	Squat-style	133.56 ± 4.78	124.06 ± 4.72	115.02 ± 5.2	134.85 ± 4.43	154.94 ± 4.20	146.66 ± 7.32	123.06 ± 4.19	179.83 ± 5.39
	*p* value	0.300	0.406	0.464	0.624	0.865	0.019	<0.001	0.648
	Cohen’s *d*	0.344	0.273	0.241	0.164	0.053	0.796	4.661	0.146
Vertical velocity of barbell (m/s)	Lunge-style	0	−0.91 ± 0.12	0	1.53 ± 0.08	0	−0.41 ± 0.17	0	0
	Squat-style	0	−0.89 ± 0.08	0	1.52 ± 0.14	0	−0.88 ± 0.14	0	0
	*p* value	—	0.727	—	0.723	—	<0.001	—	—
	Cohen’s *d*		0.191		0.091		2.98		
Distance between the two centers on the *X* axes (cm)	Lunge-style	1.42 ± 0.54	1.25 ± 0.81	1.41 ± 0.70	2.91 ± 1.50	2.06 ± 1.90	2.94 ± 2.16	4.26 ± 2.07	4.23 ± 2.56
	Squat-style	1.20 ± 0.88	1.22 ± 0.92	1.31 ± 0.83	1.53 ± 1.22	1.99 ± 1.18	3.28 ± 1.67	3.15 ± 2.57	3.44 ± 1.80
	*p* value	0.345	0.921	0.683	0.003	0.895	0.581	0.14	0.268
	Cohen’*s d*	0.311	0.035	0.132	0.996	0.043	0.173	0.483	0.349
Distance between the two centers on the *Y* axes (cm)	Lunge-style	2.42 ± 0.86	2.49 ± 1.16	2.44 ± 1.20	2.83 ± 1.53	3.44 ± 1.77	3.63 ± 1.85	3.84 ± 1.84	3.73 ± 2.17
	Squat-style	2.1 ± 1.10	2.18 ± 1.11	2.16 ± 1.12	1.68 ± 1.15	2.62 ± 1.27	3.25 ± 1.80	3.73 ± 2.36	3.32 ± 2.18
	*p* value	0.31	0.385	0.447	0.011	0.101	0.515	0.876	0.554
	Cohen’s *d*	0.330	0.272	0.240	0.834	0.521	0.208	0.053	0.189
Distance between the two centers on the *Z* axes (cm)	Lunge-style	37.27 ± 1.64	34.92 ± 1.17	30.97 ± 1.36	37.65 ± 1.06	73.30 ± 3.57	74.68 ± 2.46	72.16 ± 2.75	79.97 ± 3.03
	Squat-style	38.02 ± 1.53	36.03 ± 1.40	32.23 ± 1.18	38.63 ± 1.33	73.16 ± 2.97	75.49 ± 2.83	69.18 ± 3.13	81.30 ± 2.95
	*p* value	0.357	0.01	0.003	0.014	0.892	0.34	0.003	0.167
	Cohen’s *d*	0.471	0.871	0.980	0.828	0.042	0.308	1.021	0.444

## Data Availability

Data will be made available on request.
